# Diabetes mellitus-related hospital admissions and prescriptions of antidiabetic agents in England and Wales: an ecological study

**DOI:** 10.1186/s12902-023-01352-z

**Published:** 2023-05-06

**Authors:** Gayda Abdel Rahman AbuHammad, Abdallah Y. Naser, Loay Khaled Mohammad Hassouneh

**Affiliations:** 1 Department of Applied Pharmaceutical Sciences and Clinical Pharmacy, Faculty of Pharmacy, Isra University, Amman, Jordan; 2 Department of Respiratory Therapy, Faculty of Allied Medical Sciences, Isra University, Amman, Jordan

**Keywords:** Admissions, Diabetes mellitus, England, Prescriptions, Wales

## Abstract

**Background:**

Around 6.5% of the population in the United Kingdom has been diagnosed with diabetes. It is associated with several long-term consequences and higher hospitalization rates.

**Aim:**

To examine the profile of hospital admissions related to diabetes mellitus and the prescription rates of antidiabetic medications in England and Wales.

**Method:**

This is an ecological study that was conducted for the period between April 1999 and April 2020 using publicly available hospitalisation data in England and Wales. Hospital admission data for patients of all ages was extracted from Hospital Episode Statistics in England and the Patient Episode Database for Wales. The difference between admission rates in 1999 and 2020, as well as the difference between diabetes mellitus medication prescription rates in 2004 and 2020, were assessed using the Pearson Chi-squared test. A Poisson regression model with robust variance estimation was used to examine the trend in hospital admissions.

**Results:**

A total of 1,757,892 diabetes mellitus hospital admissions were recorded in England and Wales during the duration of the study. The hospital admission rate for diabetes mellitus increased by 15.2%. This increase was concomitant with an increase in the antidiabetic medication prescribing rate of 105.9% between 2004 and 2020. Males and those in the age group of 15–59 years had a higher rate of hospital admission. The most common causes of admissions were type 1 diabetes mellitus related complications, which accounted for 47.1% of all admissions.

**Conclusion:**

This research gives an in-depth overview of the hospitalization profile in England and Wales during the previous two decades. In England and Wales, people with all types of diabetes and related problems have been hospitalized at a high rate over the past 20 years. Male gender and middle age were significant determinants in influencing admission rates. Diabetes mellitus type 1 complications were the leading cause of hospitalizations. We advocate establishing preventative and educational campaigns to promote the best standards of care for individuals with diabetes in order to lower the risk of diabetes-related complications.

**Supplementary Information:**

The online version contains supplementary material available at 10.1186/s12902-023-01352-z.

## Background

Diabetes mellitus (DM) is a metabolic disorder characterized by hyperglycemia as a result of abnormal insulin secretion and insulin action. Individuals suffering from type II diabetes usually have insulin resistance and insulin deficiency [1]. According to the International Diabetes Federation (IDF) report for 2021, 537 million adults are living with diabetes, which is around 10% of the total population [2]. This number is predicted to rise to 643 million by 2030 and 783 million by 2045 [2]. In addition, the IDF reported that more than 75% of adults with diabetes live in low- and middle-income countries [2]. The prevalence rate of diabetes in high-income countries, middle-income countries, and low-income countries is 11.1%, 10.8%, and 5.5%, respectively [2]. In 2022, 8.75 million people worldwide were diagnosed with type 1 diabetes mellitus. One-fifth of these persons (1.9 million) reside in low- and lower-middle-income countries [3]. In 2022, 1.52 million (17%) people with T1D were younger than 20 years old, 5.56 million (64.0%) were between 20 and 59 years old, and 1.67 million (19.9%) were 60 or older [3]. Diabetes estimates for 2021 show increasing prevalence of diabetes by age. Similar trends are predicted for 2045. Adults aged 20 to 24 have the lowest prevalence (2.2% in 2021). Diabetes prevalence is anticipated to be 24.0% in individuals aged 75 to 79 in 2021 and to increase to 24.7% in 2045 [2].

In 2021, approximately 6.7 million deaths among adults aged 20–79 years were attributed to diabetes or its complications, and the direct health expenditures due to diabetes are close to one trillion United States dollars (USD) [2].

In the UK, approximately 6.5% of the population is diagnosed with diabetes [4]. According to the National Diabetes Inpatient Audit (NaDIA), 18% of acute hospital beds are occupied by individuals with diabetes, and in 11 hospital sites, 25% to 31% of inpatients have diabetes [5]. Steventon et al. reported that in England, emergency admissions among the general public increased by 42% from 4.25 million in 2006/07 to 6.02 million in 2017/18 [6]. For patients older than 85 years, emergency admissions increased by 58.9% since 2006/07. In addition, Zaccardi et al.found that between 2005 and 2014, hypoglycaemic admissions increased in England by 14.0% [7].

Rates of hospital admission for individuals with diabetes were studied in the literature, and several studies found that hospital admission in individuals with diabetes is 2–6 times higher than in people without diabetes [8, 9]. Jiang et al. reported that 30% of individuals with diabetes have 2 or more hospitalizations per year [10]. Other studies have estimated that hospital admission rates of individuals with diabetes are two to six times higher than those without diabetes [11–13]. Individuals with diabetes are more likely to be hospitalized and stay there longer if they have a low socioeconomic status, are older, are overweight, smoke cigarettes, don't exercise, or have poor control of their blood sugar [14].

A previous retrospective population-based cohort study conducted by Khalid et al. aimed to examine the rates and risk of hospitalizations in patients with type II diabetes mellitus in England and found that 60% of diabetic patients (out of 97,689) with type II diabetes had at least one hospitalization during the 4-year study period [15]. The risk of diabetes-related hospitalization increased with age, female gender, insulin use, chronic renal insufficiency, and hypoglycemia. Another observational study was conducted by Naser et al. during the period 1999–2016 to explore the trends in hospital admissions due to hypoglycemia and hyperglycemia and in the prescriptions of antidiabetic medications in England and Wales [16]. Their results showed that hospital admission rates increased by 173.0% between 1999 and 2016, for hypoglycemia, and by 147.0% for hyperglycemia. The prescription rate for all antidiabetic medications increased by 116.0% between 2004 and 2016. The rate of antidiabetic medication prescriptions increased during the same study period, with correlation coefficients of 0.94 for hypoglycemia and 0.98 for hyperglycemia, respectively.

Previous studies have focused on investigating the hospitalisation profile for specific health outcomes among patients with diabetes, such as hypoglycemia, ketoacidosis, and seizure, or a specific population, such as type 1 or type 2 diabetes mellitus patients [7, 16–20]. Therefore, this study aimed to examine the hospital admissions profile related to all types of diabetes mellitus among all age groups in England and Wales. This will help in defining the most common causes of admissions among patients with diabetes mellitus and identify high-risk populations. Our secondary objective was to examine antidiabetic medication prescribing rates.

## Method

### Study design

This is a longitudinal ecological descriptive study using public data extracted from the Hospital Episode Statistics (HES) database in England and the Patient Episode Database for Wales (PEDW) for the period between April 1999 and April 2020 [21, 22]. These two medical databases were frequently used to examine the trend of hospital admissions related to different diseases in England and Wales across different age groups [23–30]. Publicly available data from these two medical databases are at the population-level, stratified by age and gender.

### Study sources and population

Hospital admission data was collected from the HES database and the PEDW database. Hospital admissions were identified using the 10th version of the International Statistical Classification of Diseases (ICD) system. Collected data includes patient demographics, clinical diagnoses, procedures, and duration of stay. The data contains admissions of patients with diabetes mellitus from four age groups: under 15 years; 15 – 59 years; 60 – 74 years; and 75 years and above. All hospital admissions for patients with diabetes mellitus were identified using the diagnostic codes (E08 – E13). Diabetes diagnostic codes were the primary diagnosis for the patients included in this study. Prescriptions data are available from 2004 onwards. We calculate the annual hospital admission rate using mid-year population data for the period between 1999 and 2020 from the Office for National Statistics (ONS) [31].

### Statistical analysis

The number of hospital admissions for each age group was divided by the mid-year population of the same age group in the same year to produce hospital admission rates and associated 95 percent confidence intervals (CIs). Same formula was followed to calculate hospital admission rates for males and females’ patients. The difference in hospital admission rates between 1999 and 2020 was determined using the Pearson Chi-square test. A Poisson regression model with robust variance estimation was used to examine the trend in hospital admissions and the trend in medication prescribing. The confidence interval was estimated using the following equation for the population proportion: p^ + / − z* (p^(1 − p^)/n)^0.5^. SPSS software version 27 was used for all analyses (IBM Corp., Armonk, NY, USA).

## Results

### The overall hospital admissions profile

**Fig. 1:**
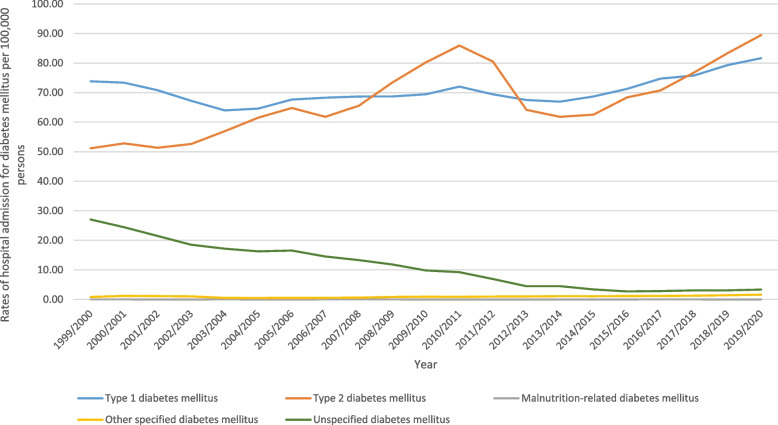
Hospital admission rates due to diabetes mellitus in England and Wales stratified by type between 1999 and 2020

During the study, from 1999 to 2020, there were a total of 1,757,892 hospital admissions in England and Wales that were linked to different diagnostic codes for diabetes and its complications. The overall rate of hospital admissions increased by 15.2% [from 152.87 (95% CI 151.81– 153.93) in 1999 to 176.05 (95% CI 174.98– 177.11) in 2020 per 100,000 persons, trend test, *p* < 0.01]. Table 1 presents the distribution of hospital admissions stratified by diabetes type. The most common admission reasons were type I diabetes mellitus (47.1%) and type II diabetes mellitus-related hospital admissions (45.1%). Figure 1 presents the rates of hospital admission rates due to diabetes mellitus between 1999 and 2020. The time series analysis for the admission rates across the study period is available in the Supplementary File 1.

**Table 1:** Distribution of hospital admissions stratified by diabetes type (three-digit diagnosis)

ICD code	Description	Percentage from total number of admissions
E10	“Type I diabetes mellitus”	47.1%
E11	“Type II diabetes mellitus”	45.1%
E12	“Malnutrition-related diabetes mellitus”	< 0.01%
E13	“Other specified diabetes mellitus”	0.7%
E14	“Unspecified diabetes mellitus”	7.2%

*ICD* International Statistical Classification of Diseases system

### The overall hospital admission rates stratified by age

**Fig. 2:**
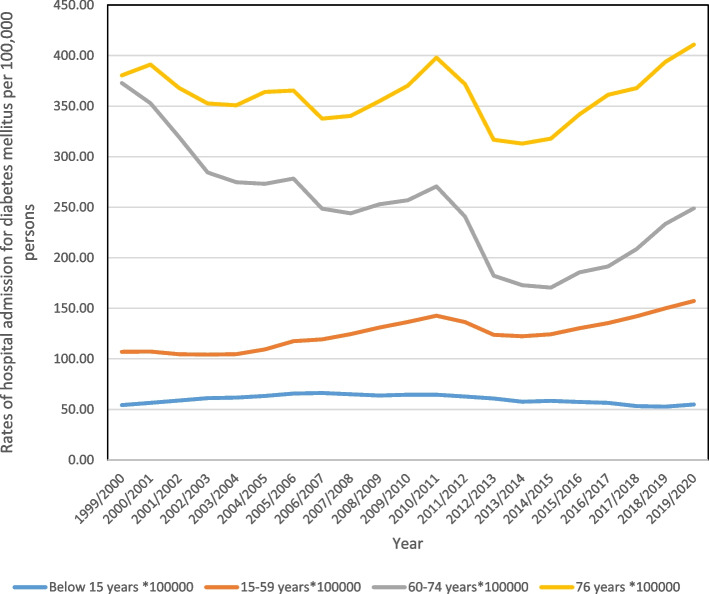
Hospital admission rates due to diabetes mellitus in England and Wales between 1999 and 2020 stratified by age group

In terms of age, patients between the ages of 15 and 59 comprised 50% of all hospital admissions (Fig. 2). The rate of hospital admissions for patients under 15 years old increased by 1.1%, from 54.27 (95% CI 52.82–55.72) in 1999 to 54.87 (95% CI 53.47–56.27) per 100,000 persons in 2020. For patients aged 15–59, the rate of admission rose by 47.0%, from 106.96 (95% CI: 105.81–108.10) in 1999 to 157.23 (95% CI: 1.32–155.91) per 100,000 persons in 2020. The rate of admissions for people aged 60 to 74 dropped by 33.2%, from 372.80 (95% CI 368.27–377.34) in 1999 to 248.91 (95% CI 245.71–252.10) per 100,000 persons in 2020. The rate of admission for patients aged 75 and older went up by 8%, from 380.43 (95% CI: 374.33–386.53) in 1999 to 410.80 (95% CI: 405.29–416.31) per 100,000 persons in 2020.

### The overall hospital rates stratified by sex

**Fig. 3:**
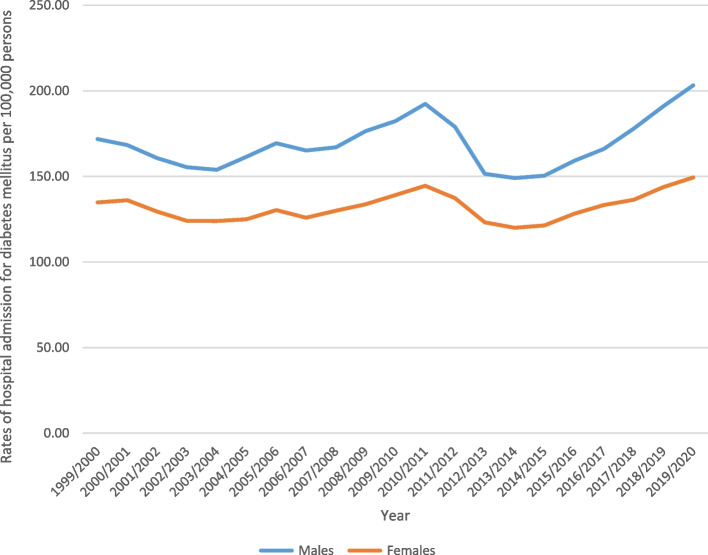
Hospital admission rates due to diabetes mellitus in England and Wales between 1999 and 2020 stratified by gender

Males contributed to 55.4% of the total number of hospital admissions, accounting for 973,814 admission episodes. Hospital admission rate among females increased by 10.8% [from 134.83 (95% CI 133.44– 136.22) in 1999 to 149.39 (95% CI 148.01– 150.76) in 2020 per 100,000 persons]. Among males, the admission rate increased by 18.3% [from 171.81 (95% CI 170.20– 173.42) in 1999 to 203.21 (95% CI 201.58– 204.83) in 2020 per 100,000 persons] (Fig. 3). For further details on the hospital admission rates stratified by type of diabetes, age groups, and sex, refer to the Supplementary File 1.

Table 2 presents the change in the hospitalisation rate stratified by type of diabetes for the period between 1999 and 2020.

**Table 2:** Percentage change in hospitalisation rate for diabetes mellitus with 95% CI

Type of diabetes mellitus	Hospitalisation rate in 1999 per 100,000 persons (95% CI)	Hospitalisation rate in 2020 per 100,000 persons (95% CI)	% Change from 1999—2020	Average change per year
All types of diabetes mellitus	152.87 (151.81– 153.93)	176.05 (174.98– 177.11)	15.2%	0.7%
“Type I diabetes mellitus”	73.81 (73.07–74.55)	81.63 (80.91–82.35)	10.6%	0.5%
“Type II diabetes mellitus”	51.15 (50.54–51.76)	89.46 (88.70–90.22)	74.9%	3.6%
“Malnutrition-related diabetes mellitus”	0.021 (0.009–0.034)	0.010 (0.002–0.018)	-52.4%	-2.5%
“Other specified diabetes mellitus”	0.83 (0.75–0.91)	1.62 (1.52–1.72)	94.7%	4.5%
“Unspecified diabetes mellitus”	27.06 (26.61–27.50)	3.33 (3.18–3.48)	-87.7%	-4.2%

### Hospital admission rates for type I diabetes mellitus

**Fig. 4:**
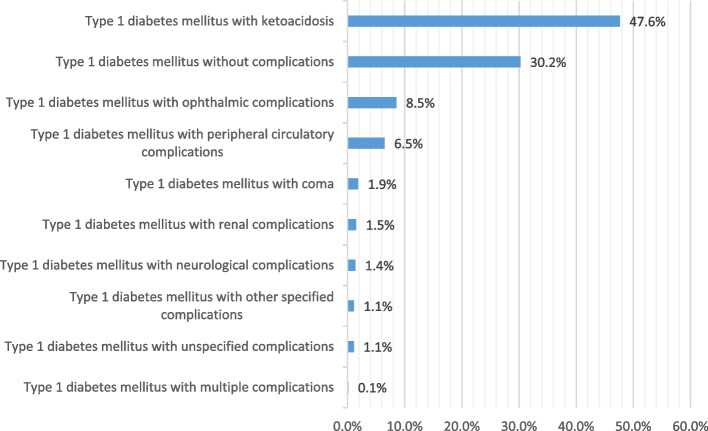
Distribution of hospital admissions for type I diabetes mellitus

**Fig. 5:**
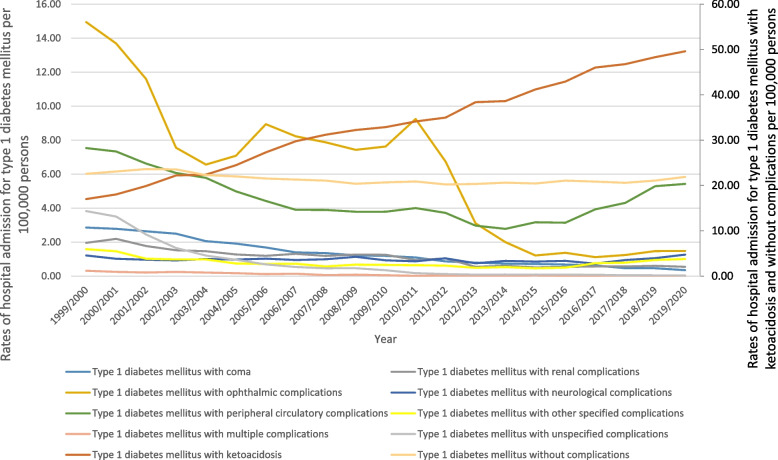
Rates of hospital admission for type I diabetes mellitus in England and Wales stratified by type between 1999 and 2020

Figure 4 shows that about half (47.6%) of hospital admissions for patients with type 1 diabetes mellitus were related to type I diabetes mellitus with ketoacidosis. The hospitalisation rate for type 1 diabetes mellitus increased by 10.6% during the study period with an average of 0.7% per year, Table 2. The rate of hospital admission for type I diabetes mellitus with ketoacidosis increased by 191.8%, and the rate of type I diabetes mellitus with neurological complications increased by 4%. However, the rate for all other complications decreased (Fig. 5). Further details on hospital admission for type I diabetes mellitus stratified by sex and age groups are available in Supplementary File 2.

### Hospital admission rates for type II diabetes mellitus

**Fig. 6:**
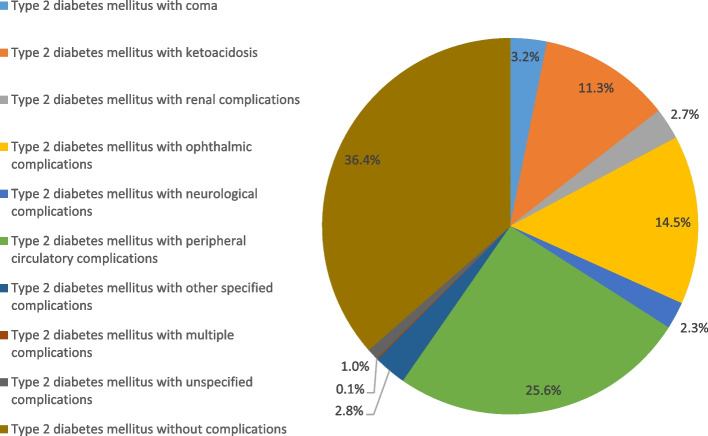
Distribution of hospital admissions for type II diabetes mellitus

Figure 6 presents the distribution of hospitalization admissions for type II diabetes mellitus patients. The hospitalisation rate for type 2 diabetes mellitus increased by 74.9% during the study period with an average of 3.6% per year, Table 2. Around 36.4% of hospitalization admissions for type II diabetes mellitus were for type II diabetes mellitus without complications, followed by type II diabetes mellitus with peripheral circulatory complications (25%), and type II diabetes mellitus with ophthalmic complications (14.5%).

**Fig. 7:**
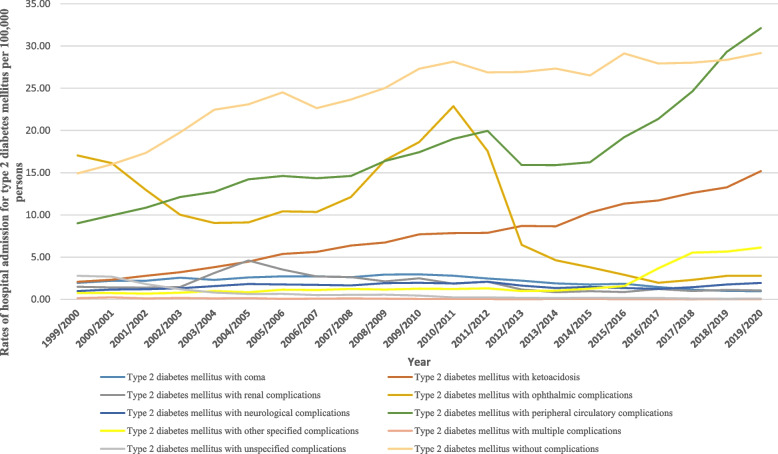
Rates hospital admission for type II diabetes mellitus in England and Wales stratified by type between 1999 and 2020

The rate of hospital admission for type II diabetes mellitus with other specified complications increased by 711.0%, followed by type II diabetes mellitus with ketoacidosis at 630.9%, and type II diabetes mellitus with peripheral circulatory complications at 256.4% (Fig. 7). Further details on hospital admission for type II diabetes mellitus stratified by sex and age groups are available in Supplementary File 2.

### Hospital admission rates for other specified diabetes mellitus

**Fig. 8:**
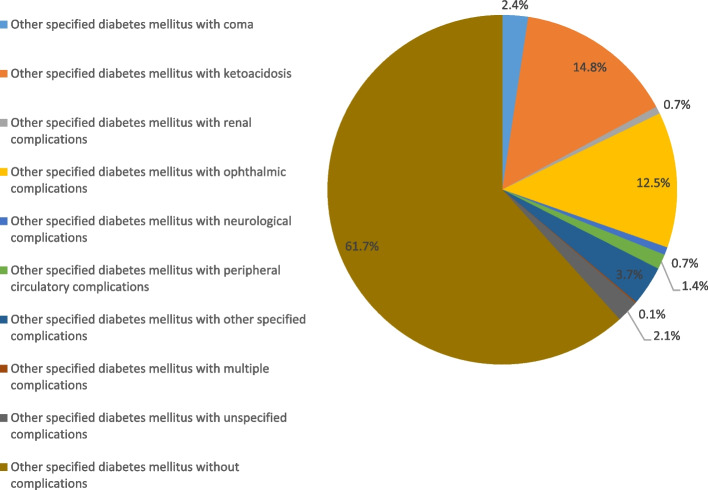
Distribution of hospital admission rates for other specified diabetes mellitus

**Fig. 9:**
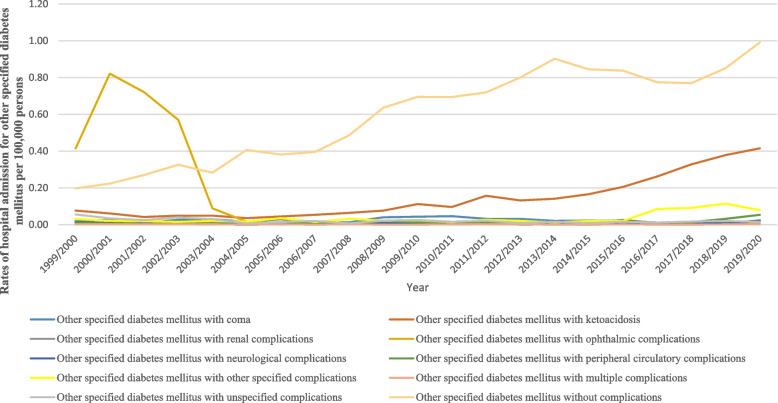
Rates of hospital admission for other specified diabetes mellitus in England and Wales stratified by type between 1999 and 2020

Figure 8 presents hospital admission rates for patients with other specified diabetes mellitus. The hospitalisation rate for other specified diabetes mellitus increased by 94.7% during the study period with an average of 4.5% per year, Table 2. The rate of hospital admission for patients with other specified diabetes mellitus without complications was the highest rate at 61.7%, followed by other specified diabetes mellitus with ketoacidosis at 14.8%, and other specified diabetes mellitus with ophthalmic complications at 15.5%. However, the hospitalization rate decreased for other specified diabetes mellitus with ophthalmic complications, other specified diabetes mellitus with unspecified complications, and other specified diabetes mellitus with multiple complications by 98.8%, 72.9%, and 12.7%, respectively (Fig. 9). Further details on hospital admission for other specified diabetes mellitus stratified by sex and age groups are available in Supplementary File 2.

### Hospital admission for unspecified diabetes mellitus

**Fig. 10:**
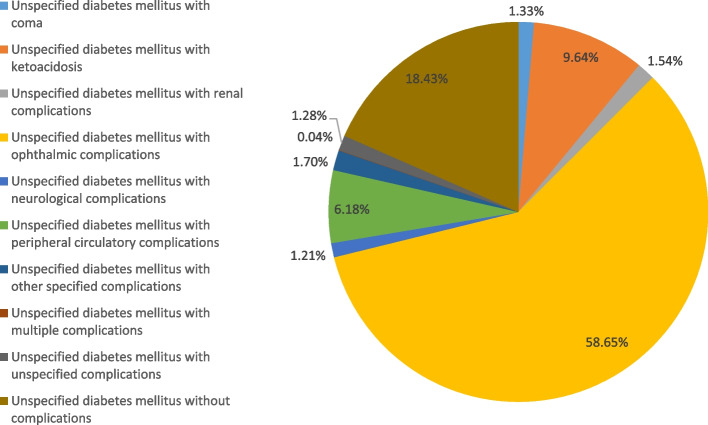
Distribution of hospital admission rates for unspecified diabetes mellitus

**Fig. 11:**
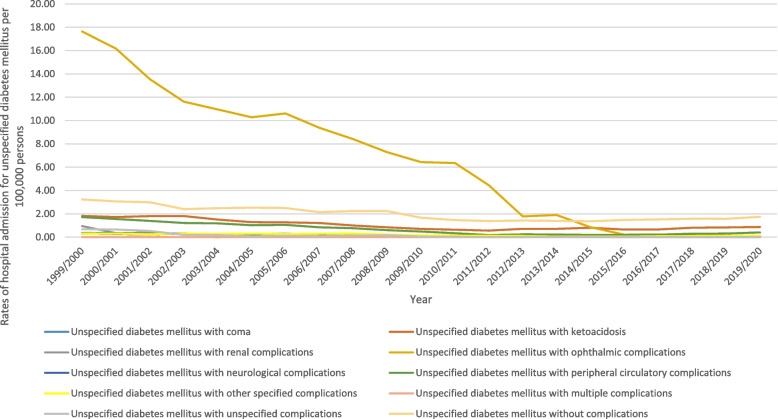
Rates of hospital admission for unspecified diabetes mellitus in England and Wales stratified by type between 1999 and 2020

Figure 10 presents the distribution of hospital admissions for unspecified diabetes mellitus. The hospitalisation rate for unspecified diabetes mellitus decreased by 87.7% during the study period with an average of 4.2% per year, Table 2. More than half of hospitalization admissions were for unspecified diabetes mellitus with ophthalmic complications (58.7%). Unspecified diabetes mellitus without complications, unspecified diabetes mellitus with ketoacidosis, and unspecified diabetes mellitus with peripheral circulatory complications contributed to 18.4%, 9.6%, and 6.2%, respectively. Figure 11 presents the rate of hospital admissions for unspecified diabetes mellitus. Further details on hospital admission for unspecified diabetes mellitus stratified by sex and age groups are available in Supplementary File 2.

### Diabetes mellitus medication prescriptions

**Fig. 12:**
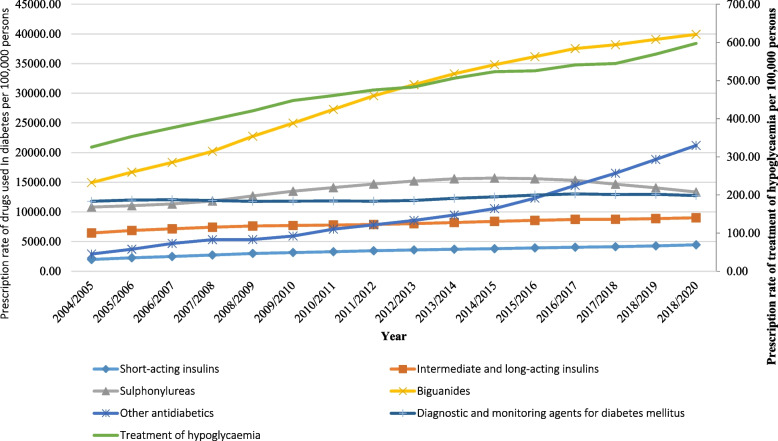
Prescription rates of diabetes mellitus medications in England and Wales between 2004 and 2020

Figure 12 presents the prescribing rate for antidiabetic medications for the period between 2004 and 2020. The diabetes mellitus medication prescription rate in England and Wales increased by 105.9% [from 49,208.37 (95% CI 49,190.03 – 49,226.71) in 2004 to 101,306.86 (95% CI 101,282.66 – 101,331.06) in 2020 per 100,000 persons, trend test, *p* < 0.01]. The time series analysis for the prescriptions rates across the study period is available in the Supplementary File 1.

Table 3 presents the change in the prescription rate for antidiabetic medications stratified by therapeutic class for the period between 2004 and 2020. The prescribing rate of biguanides increased by 167.3% [from 14,937.54 (95% CI 14,927.98—14,947.10) in 2004 to 39,928.28 (95% CI 39,915.86 – 39,940.71) in 2020 per 100,000 persons, trend test, *p* < 0.01]. Sulphonylureas prescription rate increased by 23.5% [from 10,817.13 (95% CI 10,808.80—10,825.46) in 2004 to 13,355.52 (95% CI 13,346.89 – 13,364.14) in 2020 per 100,000 persons, trend test, *p* < 0.05]. During the same period, the prescription rate for other classes of antidiabetic agents showed a much higher increase of 6.3 fold, [from 2,907.30 (95% CI 2,902.79 – 2,911.81) in 2004 to 21,203.02 (95% CI 21,192.65 – 21,213.39) in 2020 per 100,000 persons, trend test, *p* < 0.01].

**Table 3:** Percentage change in diabetes mellitus medication prescription rates from 2004 – 2020 with 95% CI

Diabetes mellitus medication	Medication prescription rates in 2004 per 100,000 persons (95% CI)	Medication prescription rates in 2020 per 100,000 persons (95% CI)	% Change from 2004—2020	Average change per year
**“Short-acting insulins”**	1986.86 **(**1983.12**–**1990.60)	4456.35 **(**4451.11**–**4461.58)	124.3%	7.8%
**“Intermediate and long-acting insulins”**	6441.32 **(**6434.74–6447.90)	9013.70 **(**9006.44–9020.97)	39.9%	2.5%
**“Sulphonylureas”**	10817.13 **(**10808.80–10825.46)	13355.52 **(**13346.89–13364.14**)**	23.5%	1.5%
**“Biguanides”**	14937.54 **(**14927.98–14947.10)	39928.28 **(**39915.86–39940.71)	167.3%	10.5%
**“Other antidiabetics”**	2907.30 **(**2902.79–2911.81)	21203.02**(**21192.65–21213.39**)**	629.3%	39.3%
**“Treatment of hypoglycaemia”**	325.46 **(**323.94–326.99)	597.39 **(**595.43–599.34)	83.5%	5.2%
**“Diagnostic and monitoring agents for diabetes mellitus”**	11792.75 **(**11784.10**–**11801.40)	12752.60 **(**12744.14**–**12761.06)	8.1%	0.5%

In terms of insulin therapy, the prescription rate for short-acting insulin increased by 1.2 fold [from 1,986.86 (95% CI 1,983.12 – 1,990.60) in 2004 to 4,456.35 (95% CI 4,451.11—4,461.58) in 2020 per 100,000 persons, trend test, *p* < 0.01]. Intermediate and long-acting insulin prescription rate increased by 39.9% [from 6,441.32 (95% CI 6,434.74 – 6,447.90) in 2004 to 9,013.70 (95% CI 9,006.44 – 9,020.97) in 2020 per 100,000 persons, trend test, *p* < 0.05].

## Discussion

The presented study showed that the hospital admission rate for diabetes mellitus increased by 15.2% over the past 21 years. This increase was concomitant with an increase in the antidiabetic medication prescribing rate of 105.9% between 2004 and 2020. In line with our results, a previous study in England and Wales found an increase of 116.0% in the dispensing of antidiabetic medications in England and Wales during the period between 2004 and 2016 [16]. The increase in antidiabetic prescription rates may reflect an increase in the prevalence of diabetes or the use of intensive antidiabetic therapy (combination therapy). According to NHSBSA Statistics (2020), the number of prescription items used primarily in the treatment of diabetes has increased every year between 2015–16 and 2019–20, where antidiabetic drugs remain the most prescribed diabetes items [5].

Previous research has focused on examining the hospitalization patterns for certain health outcomes in diabetes patients, such as hypoglycemia, ketoacidosis, and seizures, or specific populations, such as those with type 1 or type 2 diabetes mellitus patients [7, 16–20]. Our study explored all admissions related to diabetes and its complications. Several studies, found that the rate of hospitalizations due to various diabetes-related outcomes increased in the UK over the last few years [5–7, 16]. The average annual increase in the prevalence rate of diabetes mellitus in England in the past 10 years is estimated to be 7.7% and 4.0% in Wales [5, 32]. This is lower than the observed increase in the admission rate in our study, which might reflect an increase in diabetes-related complications rather than an increase in the population of patients with diabetes mellitus. According to Naser et al., between 1999 and 2016, the hospital admission rate for hyperglycaemia increased by 147.0% [16]. Emergency admissions have increased for patients aged 85 years or older by 58.9% since 2006–07. According to Zaccardi et al., hypoglycaemic admissions in England increased by 14.0% between 2005 and 2014 [7]. Several studies examined the causes of hospitalization for diabetes mellitus [33–35]. Elsayed et al. reported that the most common reason for diabetes admission was chronic complications like cardiovascular disease (47.7%) [33]. Misra et al*.* showed that 14% and 30% of admissions were related to type I and type II diabetic ketoacidosis, respectively [34]. Ismail et al. reported that risk factors like older age, obesity, and tobacco smoking were the most common causes of hospitalization for individuals with diabetes [14]. In line, Steventon et al. reported that older age (more than 85 years) was the most common reason for admission (42%) [6]. In our study, diabetes types I and 2 accounted for the majority of hospital admissions. Moreover, individuals with diabetes who develop ketoacidosis complications have a higher prevalence rate of hospital admission.

In our study, we found that older age and being male were the most common reasons for admission. Our study found that during the past two decades, the total number of hospitalisations due to diabetes among males was higher than that of females (55.4% males versus 44.6% females), which is consistent with a previous study [8]. The higher rate in males could be due to the higher prevalence of diabetes among males [8]. In addition, the National Service Framework for Diabetes revealed that the frequency of diabetes in England is higher in men than women [5]. A study conducted by Sattar et al. reported that males have a higher prevalence of diabetes because they have higher insulin resistance than women [36]. Several studies have found that lifestyle risk factors are linked to poor health outcomes and hospitalization [8, 11, 37]. Males are more likely to smoke, be physically inactive, and have poor glycemic control [8, 11, 37]. In addition, Comino et al. pointed out that males and smokers don’t comply with the primary care suggestions and proactive approach, which increases their rates of hospital admission [8].

Regarding age, our study showed that the rate of type II diabetes mellitus and other specified diabetes hospital admissions was higher among patients in the age group 15–59 (50%), followed by those aged 60–74 years at 23.8% and 75 years and older at 19.1%, while the age group under 15 years had the lower rate of hospital admission at 7.2%. Younger patients with diabetes have a higher risk of developing diabetes and being admitted to the hospital than older ones. The explanation for this might be the worse glycemic control among this age group [38]. This may be due to social characteristics and some other factors that reduce disease management at younger ages in addition to poor health habits, such as malnutrition and high intake of trans fats, low levels of physical activity, alcohol consumption, smoking, and high levels of stress [10]. In addition, Selvin and Parrinello explained this according to the differences in the pathophysiology of diabetes between older and younger adults [39].

A previous report by the National Service Framework for Diabetes showed that the prevalence of diabetes rises steeply with age: one in 20 people over the age of 65 in the UK has diabetes, and in people over the age of 85, this rises to one in five [5]. In line with this, Khalid et al. found that the risk of a diabetes-related hospitalization increased with age [15]. In addition, Fu et al. found a higher rate of type II diabetes admission at 31% in patients ≥ 65 years old [40]. Furthermore, it was found that a history of prior hospitalization is associated with re-hospitalization [40]. In addition, malignancy, insulin use, and the presence of pre-existing liver or renal disease might be other contributing factors for hospitalization at older ages [40].

In our study, about half (47.6%) of hospital admissions were related to type I diabetes mellitus with ketoacidosis, and the rate of hospital admission for type I diabetes mellitus with ketoacidosis increased by 191.8%. A previous retrospective cohort study in England showed a high rate of hospital admissions related to type I diabetes mellitus with ketoacidosis between 1998 and 2007 [41]. Zhong et al. found that the rate of ketoacidosis increased in men, patients with long-term diabetes, and patients > 35 years of age [41]. Similarly, Karges et al. found that type I diabetes mellitus patients with ketoacidosis who have a high HbA1c, a longer diabetes duration, or are adolescents have a higher incidence of hospital admission [42]. Moreover, Li et al. reported a higher rate of hospitalization admission for type I diabetes mellitus patients with ketoacidosis associated with a higher medical reimbursement rate, an uncontrolled diet, smoking, poor glycaemic control, and obesity [43, 44].

Another finding in our study showed that the other specified diabetes mellitus patients without complications have a higher rate of admission (61.7%), followed by ophthalmic complications (15.5%) and ketoacidosis (14.8%). This increased rate of admission might have been related to several risk factors associated with high blood glucose and lifestyle [45]. Ferm et al. found that high blood glucose and a longer duration of diabetes increase the incidence of having ophthalmic complications [45]. Furthermore, Zhong et al. found that individuals with diabetes who developed ketoacidosis had a higher hospitalization rate, which was associated with several risk factors such as being male, having a longer duration of disease, and receiving insulin therapy [41]. Therefore, the American Diabetes Association advised eye examination and retinal photography for those who have had diabetes for 3 to 5 years [46]. Ting et al. (2016) reported several risk factors responsible for increased admissions for individuals with diabetes and co-existing retinopathy, including older patients with a greater duration of diabetes, poor diabetic control, comorbidities, and lower socioeconomic status [47].

Our results showed that the rates for ketoacidosis without complications, and peripheral circulatory complications increased by 441.3% and 401.8%, respectively. A link was reported in the literature between peripheral circulatory disease and diabetes mellitus [48]. Shore et al. found that 25%-30% of individuals with diabetes have coronary artery revascularization [49]. The high rate of hospitalization in individuals with diabetes and peripheral circulatory complications might be related to the progression of the disease. Accordingly, Singh et al. found that poor glycemic control was associated with a higher prevalence of peripheral circulatory disease and worsened outcomes following vascular surgery or endovascular intervention [38]. In addition, the risk of these complications might increase according to lifestyle factors such as increased weight, low physical activity, tobacco use, and high levels of cholesterol and fat intake [50–52].

Several studies looked into the hospitalization-related complications of diabetes mellitus [33–35]. Elsayed et al. showed that chronic complications of diabetes mellitus were the most common reason for diabetes hospitalization, where cardiovascular disease was the most frequent (47.7%) complication [33]. In addition, Misra et al*.* showed that diabetic ketoacidosis admissions were 14% in patients with type I diabetes and 30% in patients with type II diabetes. Diabetic ketoacidosis admissions were higher in men and older patients [34].

To the best of our knowledge, this is the first study that explores the rates of diabetes mellitus-related hospitalisation and prescribing of diabetes medications in England and Wales without being restricted to specific diabetes-related outcomes (ex: hypoglycemia or hyperglycemia), specific types of diabetes, or specific age group. Our study provided detailed hospital admission rates for all types of diabetes mellitus related complications stratified by age and sex, giving a comprehensive description of the hospitalisation profile for this group of patients over a period of 21 years. Besides, we examined the prescribing pattern for all antidiabetic medications for a duration of 15 years. The limitation of our study is that it’s an ecologic study on the population-level (using aggregated data), which lacks information at the individual-level, which restricted our ability to account for readmission and adjust for important confounding factors such as age and sex. Therefore, we cannot establish causality or associations. Another limitation is that we included both diabetes and non-diabetes in the denominator population to calculate our admission rates estimates, which might have affected our findings. Therefore, our findings should be interpreted carefully. Based on our results, further studies are needed to identify other risk factors associated with diabetes complications and hospital admissions.

## Conclusion

This study explored the trend of hospital admissions related to diabetes mellitus in England and Wales during the period between 1999 and 2020. A high rate of hospital admissions for all types of diabetes and its related complications was recorded in England and Wales during the past two decades. This increase in hospital admissions was contaminant with an increase in the rate of antidiabetic medication prescriptions in England and Wales between 2004 and 2020. Males gender and people aged 15 to 59 were found to have a higher rate of diabetic hospitalization. To reduce the risk of diabetes-related complications, we recommend implementing preventive and educational campaigns to promote the optimal standards of care for individuals with diabetes. In addition, lifestyle changes should be taken into consideration and adopted to lose weight and maintain blood glucose levels at optimal levels. Further studies are required to identify other risk factors for different complications related to diabetes mellitus across different age groups.

## Supplementary Information

Additional file 1: Supplementary file 1. Figure S1. Rates of hospital admission for diabetes mellitus in England and Wales stratified by age group and type of diabetes mellitus. Figure S2. Rates of hospital admission for diabetes mellitus in England and Wales stratified by gender and type of diabetes mellitus. Figure S3. The time series analysis for the admission rates across the study period. Figure S4. The time series analysis for the prescriptions rates across the study period

Additional file 2: Supplementary file 2. Figure 1s. Hospital admission rates for type I diabetes mellitus with coma in England and Wales stratified by type between 1999 and 2020 stratified by age group. Figure 2s. Hospital admission rates for type I diabetes mellitus with coma in England and Wales stratified by type between 1999 and 2020 stratified by gender. Figure 3s. Hospital admission rates for type I diabetes mellitus with ketoacidosis in England and Wales stratified by type between 1999 and 2020 stratified by age group. Figure 4s. Hospital admission rates for type I diabetes mellitus with ketoacidosis in England and Wales stratified by type between 1999 and 2020 stratified by gender. Figure 5s. Hospital admission rates for type I diabetes mellitus with renal complications in England and Wales stratified by type between 1999 and 2020 stratified by age group. Figure 6s. Hospital admission rates for type I diabetes mellitus with renal complications in England and Wales stratified by type between 1999 and 2020 stratified by gender. Figure 7s. Hospital admission rates for type I diabetes mellitus with ophthalmic complications in England and Wales stratified by type between 1999 and 2020 stratified by age group. Figure 8s. Hospital admission rates for type I diabetes mellitus with ophthalmic complications in England and Wales stratified by type between 1999 and 2020 stratified by gender. Figure 9s. Hospital admission rates for type I diabetes mellitus with peripheral circulatory complications in England and Wales stratified by type between 1999 and 2020 stratified by age group. Figure 10s. Hospital admission rates for type I diabetes mellitus with peripheral circulatory complications in England and Wales stratified by type between 1999 and 2020 stratified by gender. Figure 11s. Hospital admission rates for type I diabetes mellitus with other specific complications in England and Wales stratified by type between 1999 and 2020 stratified by age group. Figure 12s. Hospital admission rates for type I diabetes mellitus with other specific complications in England and Wales stratified by type between 1999 and 2020 stratified by age group. Figure 13s. Hospital admission rates for type I diabetes mellitus with multiple complications in England and Wales stratified by type between 1999 and 2020 stratified by age group. Figure 14s. Hospital admission rates for type I diabetes mellitus with multiple complications in England and Wales stratified by type between 1999 and 2020 stratified by gender. Figure 15s. Hospital admission rates for type I diabetes mellitus with unspecified complications in England and Wales stratified by type between 1999 and 2020 stratified by age group. Figure 16s. Hospital admission rates for type I diabetes mellitus with unspecified complications in England and Wales stratified by type between 1999 and 2020 stratified by gender. Figure 17s. Hospital admission rates for type I diabetes mellitus without complications in England and Wales stratified by type between 1999 and 2020 stratified by age group. Figure 18s. Hospital admission rates for type I diabetes mellitus without complications in England and Wales stratified by type between 1999 and 2020 stratified by gender. Figure 19s. Hospital admission rates for type II diabetes mellitus with coma in England and Wales between 1999 and 2020 stratified by age group. Figure 20s. Hospital admission rates for type II diabetes mellitus with coma in England and Wales between 1999 and 2020 stratified by gender. Figure 21s. Hospital admission rates for type II diabetes mellitus with ketoacidosis in England and Wales between 1999 and 2020 stratified by age group. Figure 22s. Hospital admission rates for type II diabetes mellitus with ketoacidosis in England and Wales between 1999 and 2020 stratified by gender. Figure 23s. Hospital admission rates for type II diabetes mellitus with renal complications in England and Wales between 1999 and 2020 stratified by age group. Figure 24s. Hospital admission rates for type II diabetes mellitus with renal complications in England and Wales between 1999 and 2020 stratified by gender. Figure 25s. Hospital admission rates for type II diabetes mellitus with ophthalmic complications in England and Wales between 1999 and 2020 stratified by age group. Figure 26s. Hospital admission rates for type II diabetes mellitus with ophthalmic complications in England and Wales between 1999 and 2020 stratified by gender. Figure 27s. Hospital admission rates for type II diabetes mellitus with neurological complications in England and Wales between 1999 and 2020 stratified by age group. Figure 28s. Hospital admission rates for type II diabetes mellitus with neurological complications in England and Wales between 1999 and 2020 stratified by gender. Figure 29s. Hospital admission rates for type II diabetes mellitus with peripheral circulatory complications in England and Wales between 1999 and 2020 stratified by age group. Figure 30s. Hospital admission rates for type II diabetes mellitus with peripheral circulatory complications in England and Wales between 1999 and 2020 stratified by gender. Figure 31s. Hospital admission rates for type II diabetes mellitus with other specified complications in England and Wales between 1999 and 2020 stratified by age group. Figure 32s. Hospital admission rates for type II diabetes mellitus with other specified complications in England and Wales between 1999 and 2020 stratified by gender. Figure 33s. Hospital admission rates for type II diabetes mellitus with multiple complications in England and Wales between 1999 and 2020 stratified by age group. Figure 34s. Hospital admission rates for type II diabetes mellitus with multiple complications in England and Wales between 1999 and 2020 stratified by gender. Figure 35s. Hospital admission rates for type II diabetes mellitus with unspecified complications in England and Wales between 1999 and 2020 stratified by age group. Figure 36s. Hospital admission rates for type II diabetes mellitus with unspecified complications in England and Wales between 1999 and 2020 stratified by gender. Figure 37s. Hospital admission rates for type II diabetes mellitus without complications in England and Wales between 1999 and 2020 stratified by age group. Figure 38s. Hospital admission rates for type II diabetes mellitus without complications in England and Wales between 1999 and 2020 stratified by gender. Figure 39s. Rates of hospital admission for other specified diabetes mellitus with coma between 1999 and 2020 stratified by age group. Figure 40s. Rates of hospital admission for other specified diabetes mellitus with coma between 1999 and 2020 stratified by gender. Figure 41s. Rates of hospital admission for other specified diabetes mellitus with ketoacidosis between 1999 and 2020 stratified by age group. Figure 42s. Rates of hospital admission for other specified diabetes mellitus with ketoacidosis between 1999 and 2020 stratified by gender. Figure 43s. Rates of hospital admission for other specified diabetes mellitus with renal complications between 1999 and 2020 stratified by age group. Figure 44s. Rates of hospital admission for other specified diabetes mellitus with renal complications between 1999 and 2020 stratified by gender. Figure 45s. Rates of hospital admission for other specified diabetes mellitus with ophthalmic complications between 1999 and 2020 stratified by age group. Figure 46s. Rates of hospital admission for other specified diabetes mellitus with ophthalmic complications between 1999 and 2020 stratified by gender. Figure 47s. Rates of hospital admission for other specified diabetes mellitus with neurological complications between 1999 and 2020 stratified by age group. Figure 48s. Rates of hospital admission for other specified diabetes mellitus with neurological complications between 1999 and 2020 stratified by gender. Figure 49s. Rates of hospital admission for other specified diabetes mellitus with peripheral circulatory complications between 1999 and 2020 stratified by age group. Figure 50s. Rates of hospital admission for other specified diabetes mellitus with peripheral circulatory complications between 1999 and 2020 stratified by gender. Figure 51s. Rates of hospital admission for other specified diabetes mellitus with other specified complications between 1999 and 2020 stratified by age group. Figure 52s. Rates of hospital admission for other specified diabetes mellitus with other specified complications between 1999 and 2020 stratified by gender. Figure 53s. Rates of hospital admission for other specified diabetes mellitus with multiple complications between 1999 and 2020 stratified by age group. Figure 54s. Rates of hospital admission for other specified diabetes mellitus with multiple complications between 1999 and 2020 stratified by age gender. Figure 55s. Rates of hospital admission for other specified diabetes mellitus with unspecified complications between 1999 and 2020 stratified by age group. Figure 56s. Rates of hospital admission for other specified diabetes mellitus with unspecified complications between 1999 and 2020 stratified by gender. Figure 57s. Rates of hospital admission for other specified diabetes mellitus without complications between 1999 and 2020 stratified by age group. Figure 58s. Rates of hospital admission for other specified diabetes mellitus without complications between 1999 and 2020 stratified by gender. Figure 59s. Rates of hospital admission for unspecified diabetes mellitus with coma between 1999 and 2020 stratified by age group. Figure 60s. Rates of hospital admission for unspecified diabetes mellitus with coma between 1999 and 2020 stratified by gender. Figure 61s. Rates of hospital admission for unspecified diabetes mellitus with ketoacidosis between 1999 and 2020 stratified by age group. Figure 62s. Rates of hospital admission for unspecified diabetes mellitus with ketoacidosis between 1999 and 2020 stratified by gender. Figure 63s. Rates of hospital admission for unspecified diabetes mellitus with renal complications between 1999 and 2020 stratified by age group. Figure 64s. Rates of hospital admission for unspecified diabetes mellitus with renal complications between 1999 and 2020 stratified by gender. Figure 65s. Rates of hospital admission for unspecified diabetes mellitus with ophthalmic complications between 1999 and 2020 stratified by age group. Figure 66s. Rates of hospital admission for unspecified diabetes mellitus with ophthalmic complications between 1999 and 2020 stratified by gender. Figure 67s. Rates of hospital admission for unspecified diabetes mellitus with neurological complications between 1999 and 2020 stratified by age group. Figure 68s. Rates of hospital admission for unspecified diabetes mellitus with neurological complications between 1999 and 2020 stratified by gender. Figure 69s. Rates of hospital admission for unspecified diabetes mellitus with peripheral circulatory complications between 1999 and 2020 stratified by age group. Figure 70s. Rates of hospital admission for unspecified diabetes mellitus with peripheral circulatory complications between 1999 and 2020 stratified by gender. Figure 71s. Rates of hospital admission for unspecified diabetes mellitus with other specified complications between 1999 and 2020 stratified by age group. Figure 72s. Rates of hospital admission for unspecified diabetes mellitus with other specified complications between 1999 and 2020 stratified by gender. Figure 73s. Rates of hospital admission for unspecified diabetes mellitus with multiple complications between 1999 and 2020 stratified by age group. Figure 74s. Rates of hospital admission for unspecified diabetes mellitus with multiple complications between 1999 and 2020 stratified by gender. Figure 75s. Rates of hospital admission for unspecified diabetes mellitus with unspecified complications between 1999 and 2020 stratified by age group. Figure 76s. Rates of hospital admission for unspecified diabetes mellitus with unspecified complications between 1999 and 2020 stratified by gender. Figure 77s. Rates of hospital admission for unspecified diabetes mellitus without complications between 1999 and 2020 stratified by age group. Figure 78s. Rates of hospital admission for unspecified diabetes mellitus without complications between 1999 and 2020 stratified by gender

## Data Availability

Publicly available datasets were analyzed in this study. This data can be found here: https://www.nhsbsa.nhs.uk/prescription-data/dispensing-data/prescription-cost-analysis-pca-data

## Abbreviations

UK: United Kingdom
SPSS: Statistical package for social sciences
CI: Confidence interval
NHS: National Health Services
ICD: International Statistical Classification of Diseases
