# Supplementary material for: Diabetes mellitus-related hospital admissions and prescriptions of antidiabetic agents in England and Wales: an ecological study
Source: BMC Endocr Disord. 2023 May 6;23:102. doi: 10.1186/s12902-023-01352-z (PMC10163802; doi:10.1186/s12902-023-01352-z)
Supplement: Supplementary file 1 — Additional file 1: Supplementary file 1. Figure S1. Rates of hospital admission for diabetes mellitus in England and Wales stratified by age group and type of diabetes mellitus. Figure S2. Rates of hospital admission for diabetes mellitus in England and Wales stratified by gender and type of diabetes mellitus. Figure S3. The time series analysis for the admission rates across the study period. Figure S4. The time series analysis for the prescriptions rates across the study period [file 12902_2023_1352_MOESM1_ESM.docx]

**Supplementary file 1:**

Age group (below 15 years) Age group (15-59 years)

Age group (60-74 years) Age group (75 years and above)

Age group (below 15 years) Age group (15-59 years)

Age group (60-74 years) Age group (75 years and above)

**Figure S1: Rates of hospital admission for diabetes mellitus in England and Wales stratified by age group and type of diabetes mellitus.**

Males Females

**Figure S2: Rates of hospital admission for diabetes mellitus in England and Wales stratified by gender and type of diabetes mellitus.**

**
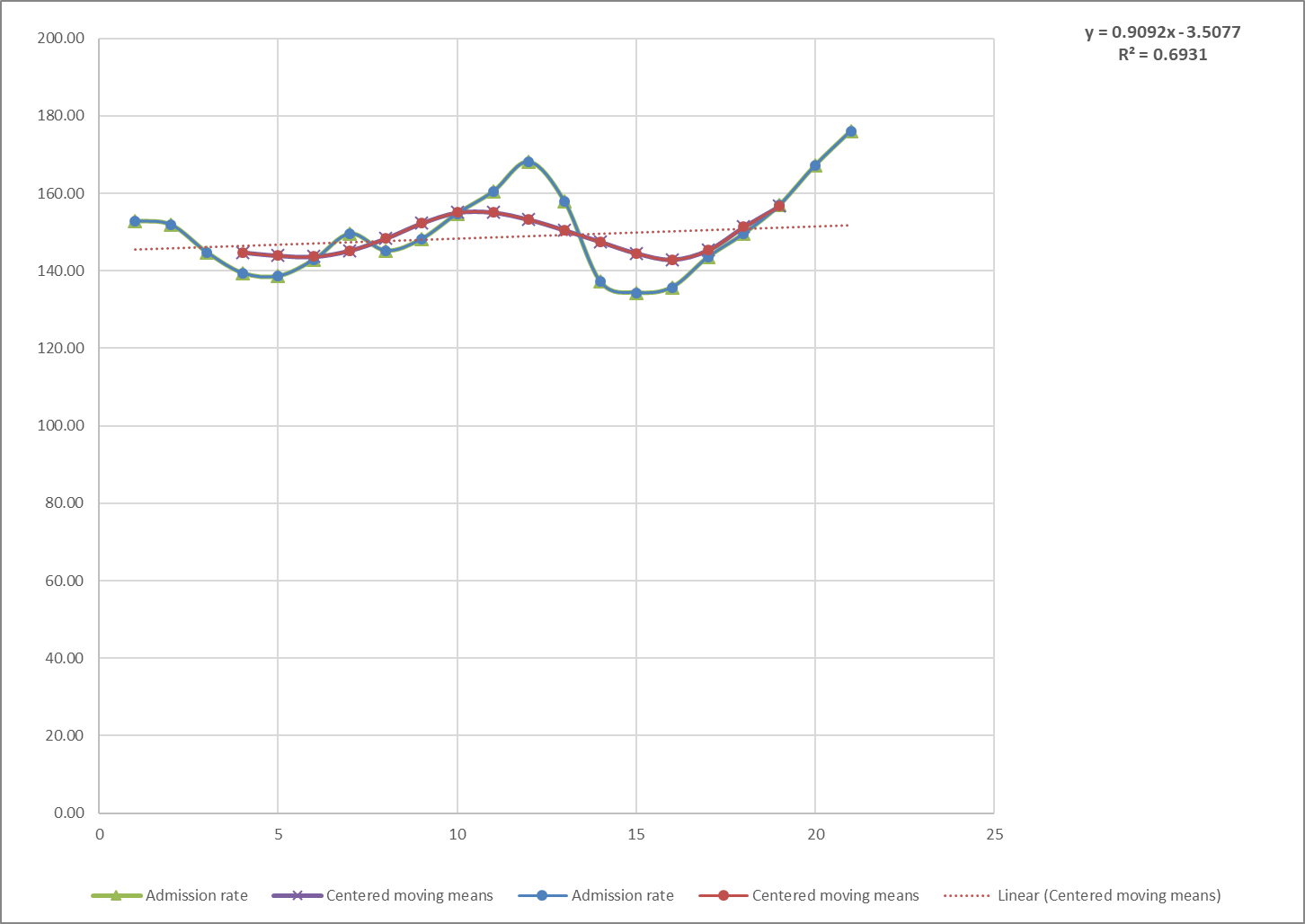
**

**Figure S3: The time series analysis for the admission rates across the study period**

**
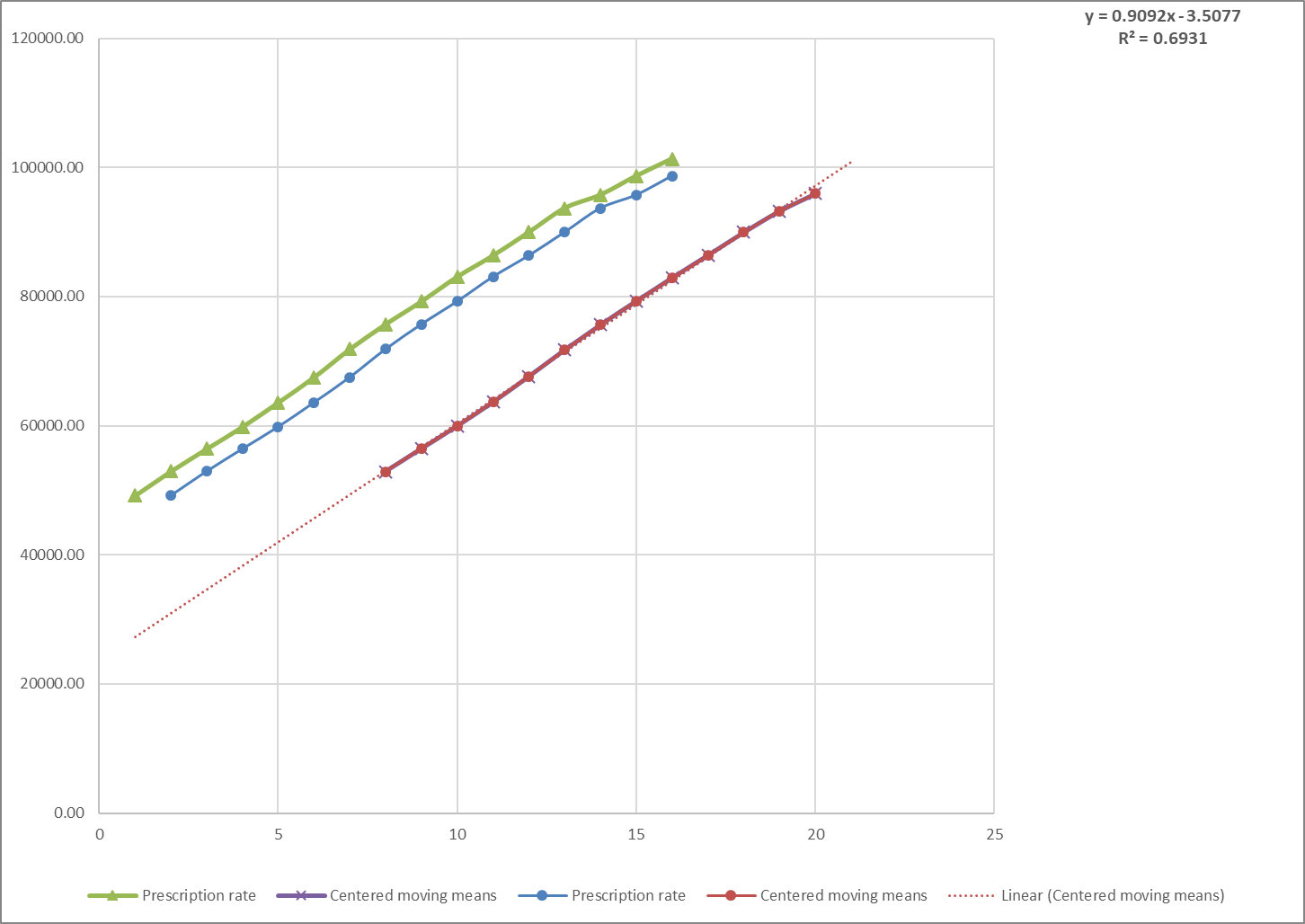
**

**Figure S4: The time series analysis for the prescriptions rates across the study period**
