# Supplementary material for: Diabetes mellitus-related hospital admissions and prescriptions of antidiabetic agents in England and Wales: an ecological study
Source: BMC Endocr Disord. 2023 May 6;23:102. doi: 10.1186/s12902-023-01352-z (PMC10163802; doi:10.1186/s12902-023-01352-z)
Supplement: Supplementary file 2 — Additional file 2: Supplementary file 2. Figure 1s. Hospital admission rates for type I diabetes mellitus with coma in England and Wales stratified by type between 1999 and 2020 stratified by age group. Figure 2s. Hospital admission rates for type I diabetes mellitus with coma in England and Wales stratified by type between 1999 and 2020 stratified by gender. Figure 3s. Hospital admission rates for type I diabetes mellitus with ketoacidosis in England and Wales stratified by type between 1999 and 2020 stratified by age group. Figure 4s. Hospital admission rates for type I diabetes mellitus with ketoacidosis in England and Wales stratified by type between 1999 and 2020 stratified by gender. Figure 5s. Hospital admission rates for type I diabetes mellitus with renal complications in England and Wales stratified by type between 1999 and 2020 stratified by age group. Figure 6s. Hospital admission rates for type I diabetes mellitus with renal complications in England and Wales stratified by type between 1999 and 2020 stratified by gender. Figure 7s. Hospital admission rates for type I diabetes mellitus with ophthalmic complications in England and Wales stratified by type between 1999 and 2020 stratified by age group. Figure 8s. Hospital admission rates for type I diabetes mellitus with ophthalmic complications in England and Wales stratified by type between 1999 and 2020 stratified by gender. Figure 9s. Hospital admission rates for type I diabetes mellitus with peripheral circulatory complications in England and Wales stratified by type between 1999 and 2020 stratified by age group. Figure 10s. Hospital admission rates for type I diabetes mellitus with peripheral circulatory complications in England and Wales stratified by type between 1999 and 2020 stratified by gender. Figure 11s. Hospital admission rates for type I diabetes mellitus with other specific complications in England and Wales stratified by type between 1999 and 2020 stratified by age group. Figure 12s. Hosp [file 12902_2023_1352_MOESM2_ESM.docx]

**Supplementary file 2**

**Figure 1s:** Hospital admission rates for type I diabetes mellitus with coma in England and Wales stratified by type between 1999 and 2020 stratified by age group

**Figure 2s:** Hospital admission rates for type I diabetes mellitus with coma in England and Wales stratified by type between 1999 and 2020 stratified by gender.

**Figure 3s:** Hospital admission rates for type I diabetes mellitus with ketoacidosis in England and Wales stratified by type between 1999 and 2020 stratified by age group

**Figure 4s:** Hospital admission rates for type I diabetes mellitus with ketoacidosis in England and Wales stratified by type between 1999 and 2020 stratified by gender

**Figure 5s:** Hospital admission rates for type I diabetes mellitus with renal complications in England and Wales stratified by type between 1999 and 2020 stratified by age group

**Figure 6s:** Hospital admission rates for type I diabetes mellitus with renal complications in England and Wales stratified by type between 1999 and 2020 stratified by gender

**Figure 7s:** Hospital admission rates for type I diabetes mellitus with ophthalmic complications in England and Wales stratified by type between 1999 and 2020 stratified by age group

**Figure 8s:** Hospital admission rates for type I diabetes mellitus with ophthalmic complications in England and Wales stratified by type between 1999 and 2020 stratified by gender

**Figure 9s:** Hospital admission rates for type I diabetes mellitus with peripheral circulatory complications in England and Wales stratified by type between 1999 and 2020 stratified by age group

**Figure 10s:** Hospital admission rates for type I diabetes mellitus with peripheral circulatory complications in England and Wales stratified by type between 1999 and 2020 stratified by gender

**Figure 11s:** Hospital admission rates for type I diabetes mellitus with other specific complications in England and Wales stratified by type between 1999 and 2020 stratified by age group

**Figure 12s:** Hospital admission rates for type I diabetes mellitus with other specific complications in England and Wales stratified by type between 1999 and 2020 stratified by age group

**Figure 13s:** Hospital admission rates for type I diabetes mellitus with multiple complications in England and Wales stratified by type between 1999 and 2020 stratified by age group

**Figure 14s:** Hospital admission rates for type I diabetes mellitus with multiple complications in England and Wales stratified by type between 1999 and 2020 stratified by gender

**Figure 15s:** Hospital admission rates for type I diabetes mellitus with unspecified complications in England and Wales stratified by type between 1999 and 2020 stratified by age group

**Figure 16s:** Hospital admission rates for type I diabetes mellitus with unspecified complications in England and Wales stratified by type between 1999 and 2020 stratified by gender

**Figure 17s:** Hospital admission rates for type I diabetes mellitus without complications in England and Wales stratified by type between 1999 and 2020 stratified by age group

**Figure 18s:** Hospital admission rates for type I diabetes mellitus without complications in England and Wales stratified by type between 1999 and 2020 stratified by gender

**Figure 19s:** Hospital admission rates for type II diabetes mellitus with coma in England and Wales between 1999 and 2020 stratified by age group

**Figure 20s:** Hospital admission rates for type II diabetes mellitus with coma in England and Wales between 1999 and 2020 stratified by gender

**Figure 21s:** Hospital admission rates for type II diabetes mellitus with ketoacidosis in England and Wales between 1999 and 2020 stratified by age group

**Figure 22s:** Hospital admission rates for type II diabetes mellitus with ketoacidosis in England and Wales between 1999 and 2020 stratified by gender

**Figure 23s:** Hospital admission rates for type II diabetes mellitus with renal complications in England and Wales between 1999 and 2020 stratified by age group

**Figure 24s:** Hospital admission rates for type II diabetes mellitus with renal complications in England and Wales between 1999 and 2020 stratified by gender

**Figure 25s:** Hospital admission rates for type II diabetes mellitus with ophthalmic complications in England and Wales between 1999 and 2020 stratified by age group

**Figure 26s:** Hospital admission rates for type II diabetes mellitus with ophthalmic complications in England and Wales between 1999 and 2020 stratified by gender

**Figure 27s:** Hospital admission rates for type II diabetes mellitus with neurological complications in England and Wales between 1999 and 2020 stratified by age group

**Figure 28s:** Hospital admission rates for type II diabetes mellitus with neurological complications in England and Wales between 1999 and 2020 stratified by gender

**Figure 29s:** Hospital admission rates for type II diabetes mellitus with peripheral circulatory complications in England and Wales between 1999 and 2020 stratified by age group

**Figure 30s:** Hospital admission rates for type II diabetes mellitus with peripheral circulatory complications in England and Wales between 1999 and 2020 stratified by gender

**Figure 31s:** Hospital admission rates for type II diabetes mellitus with other specified complications in England and Wales between 1999 and 2020 stratified by age group

**Figure 32s:** Hospital admission rates for type II diabetes mellitus with other specified complications in England and Wales between 1999 and 2020 stratified by gender

**Figure 33s:** Hospital admission rates for type II diabetes mellitus with multiple complications in England and Wales between 1999 and 2020 stratified by age group

**Figure 34s:** Hospital admission rates for type II diabetes mellitus with multiple complications in England and Wales between 1999 and 2020 stratified by gender

**Figure 35s:** Hospital admission rates for type II diabetes mellitus with unspecified complications in England and Wales between 1999 and 2020 stratified by age group

**Figure 36s:** Hospital admission rates for type II diabetes mellitus with unspecified complications in England and Wales between 1999 and 2020 stratified by gender

**Figure 37s:** Hospital admission rates for type II diabetes mellitus without complications in England and Wales between 1999 and 2020 stratified by age group

**Figure 38s:** Hospital admission rates for type II diabetes mellitus without complications in England and Wales between 1999 and 2020 stratified by gender

**Figure 39s:** Rates of hospital admission for other specified diabetes mellitus with coma between 1999 and 2020 stratified by age group

**Figure 40s:** Rates of hospital admission for other specified diabetes mellitus with coma between 1999 and 2020 stratified by gender

**Figure 41s:** Rates of hospital admission for other specified diabetes mellitus with ketoacidosis between 1999 and 2020 stratified by age group

**Figure 42s:** Rates of hospital admission for other specified diabetes mellitus with ketoacidosis between 1999 and 2020 stratified by gender

**Figure 43s:** Rates of hospital admission for other specified diabetes mellitus with renal complications between 1999 and 2020 stratified by age group

**Figure 44s:** Rates of hospital admission for other specified diabetes mellitus with renal complications between 1999 and 2020 stratified by gender

**Figure 45s:** Rates of hospital admission for other specified diabetes mellitus with ophthalmic complications between 1999 and 2020 stratified by age group

**Figure 46s:** Rates of hospital admission for other specified diabetes mellitus with ophthalmic complications between 1999 and 2020 stratified by gender

**Figure 47s:** Rates of hospital admission for other specified diabetes mellitus with neurological complications between 1999 and 2020 stratified by age group

**Figure 48s:** Rates of hospital admission for other specified diabetes mellitus with neurological complications between 1999 and 2020 stratified by gender

**Figure 49s:** Rates of hospital admission for other specified diabetes mellitus with peripheral circulatory complications between 1999 and 2020 stratified by age group

**Figure 50s:** Rates of hospital admission for other specified diabetes mellitus with peripheral circulatory complications between 1999 and 2020 stratified by gender

**Figure 51s:** Rates of hospital admission for other specified diabetes mellitus with other specified complications between 1999 and 2020 stratified by age group

**Figure 52s:** Rates of hospital admission for other specified diabetes mellitus with other specified complications between 1999 and 2020 stratified by gender

**Figure 53s:** Rates of hospital admission for other specified diabetes mellitus with multiple complications between 1999 and 2020 stratified by age group

**Figure 54s:** Rates of hospital admission for other specified diabetes mellitus with multiple complications between 1999 and 2020 stratified by age gender

**Figure 55s:** Rates of hospital admission for other specified diabetes mellitus with unspecified complications between 1999 and 2020 stratified by age group

**Figure 56s:** Rates of hospital admission for other specified diabetes mellitus with unspecified complications between 1999 and 2020 stratified by gender

**Figure 57s:** Rates of hospital admission for other specified diabetes mellitus without complications between 1999 and 2020 stratified by age group

**Figure 58s:** Rates of hospital admission for other specified diabetes mellitus without complications between 1999 and 2020 stratified by gender

**Figure 59s:** Rates of hospital admission for unspecified diabetes mellitus with coma between 1999 and 2020 stratified by age group

**Figure 60s:** Rates of hospital admission for unspecified diabetes mellitus with coma between 1999 and 2020 stratified by gender

**Figure 61s:** Rates of hospital admission for unspecified diabetes mellitus with ketoacidosis between 1999 and 2020 stratified by age group

**Figure 62s:** Rates of hospital admission for unspecified diabetes mellitus with ketoacidosis between 1999 and 2020 stratified by gender

**Figure 63s:** Rates of hospital admission for unspecified diabetes mellitus with renal complications between 1999 and 2020 stratified by age group

**Figure 64s:** Rates of hospital admission for unspecified diabetes mellitus with renal complications between 1999 and 2020 stratified by gender

**Figure 65s:** Rates of hospital admission for unspecified diabetes mellitus with ophthalmic complications between 1999 and 2020 stratified by age group

**Figure 66s:** Rates of hospital admission for unspecified diabetes mellitus with ophthalmic complications between 1999 and 2020 stratified by gender

**Figure 67s:** Rates of hospital admission for unspecified diabetes mellitus with neurological complications between 1999 and 2020 stratified by age group

**Figure 68s:** Rates of hospital admission for unspecified diabetes mellitus with neurological complications between 1999 and 2020 stratified by gender

**Figure 69s:** Rates of hospital admission for unspecified diabetes mellitus with peripheral circulatory complications between 1999 and 2020 stratified by age group

**Figure 70s:** Rates of hospital admission for unspecified diabetes mellitus with peripheral circulatory complications between 1999 and 2020 stratified by gender

**Figure 71s:** Rates of hospital admission for unspecified diabetes mellitus with other specified complications between 1999 and 2020 stratified by age group

**Figure 72s:** Rates of hospital admission for unspecified diabetes mellitus with other specified complications between 1999 and 2020 stratified by gender

**Figure 73s:** Rates of hospital admission for unspecified diabetes mellitus with multiple complications between 1999 and 2020 stratified by age group

**Figure 74s:** Rates of hospital admission for unspecified diabetes mellitus with multiple complications between 1999 and 2020 stratified by gender

**Figure 75s:** Rates of hospital admission for unspecified diabetes mellitus with unspecified complications between 1999 and 2020 stratified by age group

**Figure 76s:** Rates of hospital admission for unspecified diabetes mellitus with unspecified complications between 1999 and 2020 stratified by gender

**Figure 77s:** Rates of hospital admission for unspecified diabetes mellitus without complications between 1999 and 2020 stratified by age group

**Figure 78s:** Rates of hospital admission for unspecified diabetes mellitus without complications between 1999 and 2020 stratified by gender
